# Impact of C-reactive protein test results on evidence-based decision-making in cases of bacterial infection

**DOI:** 10.1186/1471-2431-12-140

**Published:** 2012-09-03

**Authors:** Mona Nabulsi, Abeer Hani, Maria Karam

**Affiliations:** 1 Department of Pediatrics and Adolescent Medicine, American University of Beirut Medical Center, P.O. Box: 113-6044/C8, Beirut, Lebanon

**Keywords:** C-reactive protein, Neonatal sepsis, Bacterial infection, Diagnostic accuracy

## Abstract

**Background:**

C-reactive protein (CRP) is widely used to detect bacterial infection in children. We investigated the impact of CRP test results on decision-making and summarized the evidence base (EB) of CRP testing.

**Methods:**

We collected information from the hospital records of 91 neonates with suspected sepsis and of 152 febrile children with suspected infection on the number of ordered CRP tests, the number of EB-CRP tests, and the impact of the test results on decision-making. CRP diagnostic accuracy studies focusing on pediatric infections were reviewed critically. The main outcomes were the proportion of CRP tests that were EB and the proportion of tests that affected decision-making. A secondary outcome was the overall one-year expenditure on CRP testing.

**Results:**

The current EB for CRP testing in pediatric infections is weak and suggests that CRP is of low diagnostic value. Approximately 54.8% of tests performed for suspected neonatal sepsis and 28% of tests performed for other infections were EB; however, the results of only 12.9% of neonatal sepsis tests and of 29.9% of tests on children with other infections informed decision-making. The one-year overall cost for CRP testing and related health care was $26,715.9.

**Conclusions:**

The routine ordering of CRP for children with infections is based on weak evidence. The impact of the CRP test results on decision-making is rather small, and CRP ordering may contribute to unnecessary health care expenditures. Better quality research is needed to definitively determine the diagnostic accuracy of CRP levels in children with infections.

## Background

C-reactive protein (CRP) is an acute-phase reactant that is synthesized by the liver within six hours after the onset of inflammation and tissue necrosis [1]. Its rapid synthesis, short half-life and rapid decline with recovery, together with an association between greater increases and serious bacterial infections, have made the CRP test popular. This test is often requested to help discriminate viral infections from bacterial infections or monitor the response to antibiotics [2]. The CRP level is widely used to detect bacterial infections in children with fever and in neonates with suspected sepsis [2]. However, recent evidence on the utility of the CRP test in patients with various infections suggests that there are great variations in the sensitivity, specificity, and predictive values of this test, which may compromise its diagnostic accuracy [3-5]. In addition, we have observed that despite its wide use as a diagnostic tool for several acute pediatric infections, CRP testing rarely impacted clinical decision-making. In our setting, CRP is ordered routinely on children presenting with symptoms and/or signs suggestive of acute infection as a baseline test to help discriminate viral from bacterial infection. Similarly, CRP is ordered routinely on all neonates with suspected neonatal sepsis in the neonatal intensive care unit. We therefore conducted this study to determine how often the results of CRP testing impact clinical decision-making regarding pediatric patients requiring hospital admission and to determine how often the ordering of the CRP test is evidence based.

## Methods

We conducted a retrospective chart review that included all pediatric patients admitted to the pediatric ward or the neonatal intensive care unit (NICU) of the American University of Beirut Medical Center (AUBMC) between January 1 and December 31, 2008, as identified by the Hospital Medical Records Department. AUBMC is a 420-bed university hospital that offers tertiary health care and is accredited by the Joint Commission International (JCIA). A case was reviewed in detail if it met all of the following inclusion criteria: age between 0 and 18 years; acute infection or neonatal sepsis as the admission diagnosis; and serum CRP testing performed at baseline upon admission to the emergency department (ED) or NICU. We excluded children with malignancies, congenital or acquired immune disorders, septic shock, congenital heart disease, severe chronic disease involving any organ system, rheumatic disease, collagen vascular disease, and acute or chronic hepatic disorders.

The data collected from the hospital records included age, admission diagnosis, final diagnosis, number of CRP tests performed, length of hospital stay, impact of CRP results on clinical decision-making or management (Yes/No) based on our review of physicians’ notes, additional laboratory tests or treatment preformed based on the CRP results, and the overall amount of the hospital bill at discharge. The impact of the CRP results on decision-making with respect to treatment or further laboratory testing was coded as “Yes” if clearly reflected in physicians’ notes. Otherwise, it was coded as “No”. This study was approved by the Institutional Review Board.

To assess the evidence base for CRP testing for each type of infection, we performed a systematic search for the published research available to physicians at the time at which the selected patients were admitted to the hospital (until end of 2008). We used the keywords *C-reactive protein* AND (*infection* OR *neonatal sepsis)* to search PubMed Clinical Queries for diagnostic studies (broad search) and to search the Cochrane Library for systematic reviews of diagnostic accuracy. The search was performed by one of the authors (MN) and was last updated on December 16, 2011, for studies published after 2008. Studies were reviewed in detail if the participants were children (ages 0-18 years) with acute infections in whom CRP testing was blindly and independently compared with adequate microbiologic or radiologic reference standards, and the diagnostic accuracy of CRP test results was evaluated as an outcome. Studies of neonatal sepsis were reviewed independently by two authors (MN and AH), whereas studies on other acute pediatric infections were reviewed independently by MN and MK. The quality assessment of each study was performed using the QUADAS tool [6]. Decisions on quality and study inclusion were performed independently by the same authors for the two categories of studies (MN and AH for neonatal sepsis; MN and MK for other acute pediatric infections), and these decisions were subsequently compared and discussed further among the authors.

For each type of infection, CRP testing was judged to be evidence-based if at least one valid study reported it to be of significant diagnostic value, based on likelihood ratios, sensitivity, specificity, and predictive values, for the discrimination of viral infections from bacterial infections. Studies published before the end of 2008, the year during which admissions that are under review took place, are summarized in tables ( Additional file 1 and Additional file 2). Table 1 summarizes our decision rules for the evidence base for CRP testing for each type of infection. The determination of CRP ordering as evidence-based in each case was made according to the patient’s working diagnosis at the time CRP was measured, and not based on the patient’s final diagnosis.

**Table 1:** Decision rules on the evidence-base for CRP testing in acute pediatric infections and neonatal sepsis

**Infection Type**	**Evidence-Based CRP testing**	**Non-Evidence-Based CRP testing**	**Highest available Level of Evidence**
**Fever without focus**	___	Baseline &/or follow up testing in patients with fever without focus who are >28 days of age	Level I (SR)
**Pneumonia**	___	Baseline &/or follow up testing in febrile children with respiratory symptoms and an admission diagnosis of bronchitis, bronchiolitis, asthma, or pneumonia	Level I (SR)
**Urinary tract infections (UTI)**	Baseline &/or follow up testing in febrile pyelonephritis	Baseline testing in febrile infants suspected to have UTI	Level III & IV (Cross-sectional & retrospective studies)
**Acute gastroenteritis**	___	Baseline &/or follow up testing in febrile or non-febrile acute gastroenteritis	No evidence
**Meningitis**	___	1. Baseline testing in febrile children suspected to have meningitis	Level IV (Retrospective studies)
		2. Follow up testing in febrile children with meningitis	
**Acute osteomyelitis/Septic arthritis**	Baseline &/or follow up testing in acute osteomyelitis &/or septic arthritis	___	Level IV (Case series)
**Acute appendicitis**	Baseline &/or follow up testing in acute appendicitis	___	Level IV (Cross-sectional studies)
**Acute otitis media**	___	Baseline &/or follow up testing in acute otitis media	Level II (Cross-sectional studies)
**Cellulitis**	___	Any baseline or follow up testing in patients with any of those infections	No evidence
**Acute sinusitis**			
**Tonsillitis**			
**Neonatal sepsis**	Testing on three consecutive days of a suspected sepsis episode	A single determination of CRP	Level I (SR)

### Statistical analysis

We summarized the variables of interest using the means and standard deviations, medians, ranges, and proportions. The main outcome measures were the proportion of ordered CRP tests that were supported by evidence and the proportion of tests that resulted in a change in management, such as the requesting of further tests or the stopping or starting of antibiotics. A secondary outcome was the overall one-year expenditure on CRP tests. We used the Statistical Package for Social Science (SPSS version 18, Chicago, IL) for data analysis.

## Results

Overall, we screened 2250 hospital records (nursery = 1485; pediatric ward = 765). Of these, 243/2250 (10.8%) hospital admissions met our inclusion criteria: 91/243 (37.4%) from the NICU and 152/243 (62.6%) from the pediatric ward. These cases constituted 6.1% (91/1485) of nursery admissions and 19.9% (152/765) of overall ward admissions, respectively. A total of 517 CRP tests were performed during one year: 254/517 (49.1%) for the ward patients and 263/517 (50.9%) for the NICU patients. The baseline characteristics of the NICU and ward patients and their values of the CRP-related variables are detailed in Tables 2 and 3, respectively.

**Table 2:** Baseline characteristics of NICU and ward patients

	**NICU**	**Ward**
**Number of cases**	91	152
**Male Gender N (%)**	38 (41.8%)	75 (49.3%)
**Gestational Age (weeks)**	Mean (SD) = 34.6 (4.1)	___
	Median = 35	
	Range = 25-42	
**Age (years)**	___	Mean (SD) = 3.0 (3.2)
		Median = 1.5
		Range = 0.01-12.1
**Admission Diagnosis N (%)**	Sepsis = 91 (100%)	Bacterial = 123 (80.9%)
		Non-bacterial = 29 (19.1%)
**Final Diagnosis N (%)**	Sepsis = 44 (48.4%)	Bacterial = 69 (45.4%)
	Prematurity = 21 (23.1%)	Non-bacterial = 83 (54.6%)
	Viral = 7 (7.7%)	
	Other = 19 (20.9%)	

**Table 3:** NICU and ward patients’ CRP-related outcomes and costs

**CRP-related variables**	**NICU**	**Ward**
**Baseline CRP (mg/L)**	Mean (SD) = 5.5 (10.1)	Mean (SD) = 65.9 (85.8)
	Median = 1.6	Median = 31.2
	Range = 0.1 – 68.4	Range = 0.4-512.2
**Total No. of CRP tests requested/case**	Mean (SD) = 2.9 (2.7)	Mean = 1.7 (1.0)
	Median = 2	Median = 1.0
	Range = 1-12	Range = 1 - 6
**Overall No. of EB-CRP tests (%)**	144/263 (54.8%)	71/254 (28%)
**Overall No. of clinical decisions affected by CRP (%)**	34/263 (12.9%)	76/254 (29.9%)
**CRP overall cost/year ($)**	12,799.3	12,517
**CRP overall cost/case ($)**	Mean (SD) = 140.7 (131.7)	Mean (SD) = 82.3 (48.4)
	Median = 97.3	Median = 49.3
	Range = 48.7 – 584.0	Range = 48.7 – 292.0
**% Hospital bill due to CRP**	1.1%	3.34%
**Non-EB CRP overall cost/year ($)**	5986.0	8868.7
**Non-EB CRP overall cost/case ($)**	Mean (SD) = 65.8 (64.6)	Mean (SD) = 58.3 (46.6)
	Median = 48.7	Median = 48.7
	Range = 0.0 – 292.0	Range = 0.0 – 292.0
**% CRP cost due to non-EB tests/year**	46.8%	70.8%
**Cost of additional health care due to non-EB CRP/case ($)**	___	Mean (SD) = 44.7 (262.8)
		Median = 0.0
		Range = 0.0 – 2354.7
**Overall cost due to non-EB CRP and additional health care/year ($)**	5986.0	15,662
**% Hospital bill due to non-EB CRP and additional costs/year**	1.6%	4.2%

No.: number; EB: evidence-based; non-EB: non-evidence-based.

For the 91 NICU admissions, 263 CRP tests were performed during the initial work up of suspected neonatal sepsis episodes. Of these, 144/263 (54.8%) were serial CRP tests and hence were considered evidence based (EB), whereas the remaining 119/263 (45.2%) tests were single tests and were considered non-evidence based (non-EB). The mean (SD) number of CRP tests performed per neonate was 2.9 (2.7), with a range of 1-12 tests. Among all of these CRP tests, only 34/263 (12.9%) had an impact on clinical decision-making in terms of continuing or stopping antibiotics. In the remaining cases, treatment decisions were based on culture results and/or the baby’s clinical picture, irrespective of the value of CRP.

For the 152 children admitted to the pediatric ward, the admission diagnoses were pneumonia (40), acute gastroenteritis (28), bacteremia (25), urinary tract infection (13), acute otitis media (10), meningitis (7), soft tissue/bone/joint infections (4), acute sinusitis (4), appendicitis (3), tonsillitis (2), and other miscellaneous infections (16). There was an evidence base for ordering CRP in the ED for 40/152 (26.3%) cases. The mean (SD) number of CRP tests performed per child during the hospital stay was 1.7 (1.0), with a range of 1-6 tests. Of the 254 CRP tests performed during the hospital stays of these patients, 178 (70.1%) failed to inform decision-making and/or resulted in further unnecessary additional health care.

Regarding the cost, the overall charges for the 517 CRP tests performed during one year was $25,316.3. In the NICU, the overall CRP charges were $12,799.3, with a mean (SD) of $140.7 (131.7) per neonate. Approximately $5986 (46.8%) of the total CRP charges in the NICU was spent on tests that were unsupported by evidence. When the impact of CRP testing on decision-making was taken into consideration, the overall cost of the 229/263 (87.1%) CRP tests that failed to inform decision-making was estimated at $11,148.2.

Regarding ward admissions, the overall CRP charges were $12,517 with a mean (SD) of $82.3 (48.4) per patient. Tests that were unsupported by evidence were estimated to cost $8868.7 (70.9% of total ward CRP charges). Non-EB testing resulted in the further ordering of laboratory or radiologic tests, additional antibiotic treatment, or the prolongation of the hospital stay for some cases, thus leading to additional charges amounting to $6793.3. When the impact of CRP testing on clinical decision-making was considered by including the CRP tests that failed to inform decision-making and/or resulted in further unnecessary additional health care, the expenditure on CRP testing increased to $15,567.7. As a result, our one-year charges for routine CRP testing in the NICU and for non-EB testing for other pediatric infections amounted to $26,715.9.

## Discussion

We investigated the impact of routine CRP ordering on clinical decision-making in hospitalized febrile children and neonates with suspected neonatal sepsis, the evidence base for such testing, and the one-year direct medical costs due to this practice. We found that routine CRP ordering for diagnostic purposes for these infections failed to inform decision-making in the majority of cases while inflating hospital bills by $26,715.9. Only 12.9% of CRP tests performed on neonates with suspected neonatal sepsis and 29.9% of tests performed on febrile children with suspected bacterial infections informed decision-making. Moreover, the majority of the ordered CRP tests did not have a solid evidence base to support their use as diagnostic tools for the accurate detection of bacterial infections. Almost half of CRP tests ordered during the initial workup of neonatal sepsis and about three-quarters of tests performed to investigate or follow-up children with bacterial infections were non-EB. This practice is most likely influenced by the rapidly increasing literature on the utility of CRP levels for different infections in children and neonates. However, recent systematic reviews have questioned the diagnostic accuracy of CRP for serious bacterial infections in febrile children [3-5], pneumonia [7,8], and neonatal sepsis [4,9]. Cross-sectional, retrospective and case series studies, which provide a lower level of evidence, suggest that CRP is of low diagnostic value for the diagnosis of several other infections, such as febrile urinary tract infections [10-13], acute febrile gastroenteritis [14], acute appendicitis [15], acute otitis media [16,17], community-acquired pneumonia [18], and meningitis [19]. However, other studies suggest that the CRP level may be of prognostic value in patients with acute osteomyelitis or septic arthritis [20-23] and in differentiating simple from perforated appendicitis [24]. Two old retrospective studies reported high sensitivities and negative predictive values for CRP in differentiating bacterial from viral meningitis [25] and other central nervous system infections [26]. In neonatal sepsis, a single CRP value was found to be a poor predictor of sepsis [27,28], whereas serial CRP measurements were good predictors of late-onset sepsis (LOS) in very-low-birth-weight neonates, whether performed as single tests or in combination with tests of the interleukin-6 (IL-6) level [29]. Moreover, serial CRP values were shown to be helpful in guiding the duration of antibiotic therapy in two other studies [30,31]. It should be noted, however, that all previous studies varied in their choice of the optimal CRP cutoff value, which adds to the controversy regarding the diagnostic benefit of the CRP level for pediatric infections.

This study is the first to examine the impact of CRP testing on clinical decision-making and to evaluate the evidence base for the utility of this test for different infections in children. Despite this study’s limitations, we were able to demonstrate the small contribution that this test makes to the diagnosis of bacterial infections in children and the impressive increase in hospital bills due to its routine ordering. However, this study suffers from several limitations. First, its retrospective design may introduce bias in accurately capturing the treating physician’s rationale behind CRP ordering and its effect on the management of patients if the rationale or effects were inadequately recorded in the patients’ medical charts. This bias was kept to a minimum by our careful scrutiny of the written medical orders and the progress notes pertaining to each CRP test. Second, although the classification of CRP tests as EB or non-EB was based on the highest level of evidence available at the time of admission, most of this evidence came from studies with a low level of evidence, such as retrospective studies or case series, when systematic reviews were unavailable. Third, we found great variation in the optimal CRP cutoff level among studies, even for the same type of infection. However, these limitations make our calculations of the proportion of evidence-based tests and of the extra charges due to CRP ordering in our setting an under-estimate of the reality. The main strength of this study is the use of explicit criteria to classify CRP test ordering as EB or non-EB based on a critical review of the literature.

Overutilization of laboratory testing is common in hospital practices and has been attributed to the defensive behavior of physicians, a lack of experience, uncertainty, “routine” practice, a lack of awareness of the associated costs, the use of protocols and guidelines, and other factors [32]. Inappropriate testing may increase patient anxiety, inflate health care costs, waste health resources, and affect the quality of care. Unfortunately, interventions aiming at improving appropriate laboratory ordering have been unsuccessful [32], further adding to the complexity of the problem.

## Conclusion

In conclusion, routine CRP ordering for the detection of bacterial infection in sick children and neonates needs further scrutiny by practicing physicians. The evidence in support of the diagnostic utility of CRP levels for pediatric infections is weak and is mostly based on studies of low levels of evidence. The CRP test results seem to have a small impact on decision-making and may contribute to the unnecessary elevation of health care expenditures. Better quality studies are needed to address the utility of CRP testing for pediatric infections and to define a consistent optimal cutoff value that can discriminate bacterial from viral infections or other diseases.

### Ethical approval

The study was approved by the Institutional Review Board of the American University of Beirut.

## Abbreviations

CRP, C-reactive protein; EB, Evidence base(d); NICU, Neonatal intensive care unit; ED, Emergency department; SD, Standard deviation; SR, Systematic review; UTI, Urinary tract infection.

## Competing interests

The authors declare that they have no competing interest.

## Authors’ contributions

MN is the lead investigator responsible for the conception and design of the study; the literature search and summary; the appraisal; the collection, analysis, and interpretation of the data; and the writing and revision of the manuscript. MN is the guarantor for this study. AH and MK participated in the literature search, appraisal, summary, and data collection. All authors approved the final version of this manuscript.

## Funding

This research received no specific grant from any funding agency in the public, commercial or not-for-profit sectors.

## Pre-publication history

The pre-publication history for this paper can be accessed here:

http://www.biomedcentral.com/1471-2431/12/140/prepub

## Supplementary Material

Additional file 1 **APPENDIX I.Summary of studies on the utility of CRP in pediatric infections.** Click here for file

Additional file 2 **APPENDIX II.Summary of studies on the utility of CRP in neonatal sepsis.** Click here for file
