# Supplementary material for: Impact of C-reactive protein test results on evidence-based decision-making in cases of bacterial infection
Source: BMC Pediatr. 2012 Sep 3;12:140. doi: 10.1186/1471-2431-12-140 (PMC3457842; doi:10.1186/1471-2431-12-140)
Supplement: Additional file 1 — APPENDIX I.Summary of studies on the utility of CRP in pediatric infections. [file 1471-2431-12-140-S1.doc]

**APPENDIX I: Summary of studies on the utility of CRP in pediatric infections.**

| **Study author (Year)** | **Type of study** | **Subjects** | **Interventions** | **Outcomes** | **Findings** | **Quality &**  **Comments** |
| --- | --- | --- | --- | --- | --- | --- |
| **Fever without focus**  Sanders, et al.^[3]^ (2008) | **SR** | Infants & children presenting with fever, excluding inpatients | **I:** Serum CRP  **C:** Reference standard of microbiologic diagnosis | -Serious bacterial infection (SBI) vs benign bacterial/non-bacterial infection  - Bacterial vs non-bacterial infection | - For SBI vs benign bacterial/non bacterial infection: CRP pooled sensitivity 77%, specificity 79%, LR+ 3.64, LR^-^ 0.29  - For bacterial vs nonbacterial infection: sensitivity between 22-58%, specificity 86-96%, LR+ 3.2-13.3, LR^-^ 0.4-0.8 | -Valid review; used QUADAS to assess studies  - For bacterial vs non bacterial infection, findings were generated from 3 studies that could not be pooled together  - Variable CRP cutoff points were used in the different studies |
| **Pneumonia**  **1)** Flood, et al.^[7]^  (2008)  **2)** van der Meer, et al.^[8]^ (2005)  **3)** Toikka, et al.^[18]^ (2000) | **1) SR**  **2) SR**  **3) C-S** | **1)** Acutely ill children 1m-18 yrs  **2)**Adults & children with radiological pneumonia  **3)**Children hospitalized with radiological community-acquired pneumonia | **1) I:** Serum CRP  C:CXR, clinical, microbiologic criteria  **2)** **I:** Serum CRP  **C:** CXR or reference microbiologic workup  **3)** **I:** Serum PCT, CRP, IL-6  **C:** panel of bacterial & viral detection tests | **1)** Differentiate bacterial from non-bacterial pneumonia  **2)** -Presence or absence of pneumonia  - Bacterial vs viral pneumonia  **3)** Bacterial vs viral pneumonia | **1)** - OR for bacterial pneumonia is 2.5 if CRP >35-60 mg/L; LR+ 0.65  - CRP>40-60 mg/L weakly predicts bacterial pneumonia  **2)** - Adults: For detecting infiltrates on CXR, accuracy of CRP =o.8; for detecting bacterial etiology, studies were variable and did not meet inclusion criteria  - Children: Insufficient evidence  **3)** Higher PCT & CRP  values but not IL-6 in bacterial pneumonia, but overlapping with viral pneumonia values | **1)** - Valid review  - Studies of good quality but significantly heterogeneous  **2)** - Valid review; study quality assessed using the guidelines of Cochrane Methods Group on SRs of screening & diagnostic tests  - No evidence to support use of CRP in diagnosing pneumonia of bacterial etiology  **3)** - QUADAS scale: Yes for 11/13 elements  - PCT, CRP & IL-6 cannot discriminate bacterial from viral pneumonia |
| **Pyelonephritis**  **1)** Lin, et al.^[10]^ (2000)  **2)** Pecile et al.^[11]^  (2004)  **3)** Garin et al.^[12]^  (2007)  **4)** Huang, et al.^[13]^ (2007) | **1) C-S**  **2)** **C-S**  **3)** **R-S**  **4)** **R-S** | **1)** Febrile infants <8 weeks of age  **2)** Children with febrile UTI 1m-13 yrs  **3)** Children with febrile UTI < 2yrs of age  **4)** Children with febrile UTI, fever >2 days | **1) I:** CRP & other laboratory parameters  **C:** Suprapubic urine culture  **2) I:** Serum PCT, CRP  **C:** DMSA scan  **3) I:** CRP & other laboratory parameters  **C:** DMSA scan  **4) I:** CRP, urine culture, other laboratory parameters  **C:** DMSA scan | **1)** Presence or absence of UTI  **2)** – Presence or absence of APN  - Accuracy in detecting renal scars  **3)** Differentiating upper vs lower UTI  **4)** Prediction of APN in febrile UTI | **1)** CRP>20mg/L: sensitivity 59%, specificity 90%, LR+ 5.9, LR^-^ 0.45  **2)** –PCT ≥ 0.8ng/mL had sensitivity 83.3%, specificity 93.6% in predicting APN  - CRP ≥20 mg/L had sensitivity 94.4%, specificity 31.9% in predicting APN  - PCT & CRP levels correlated significantly with severity of renal injury initially  **3)** CRP >0.5µg/ml had sensitivity of 100%, specificity 8%, accuracy 48% in diagnosing APN  **4)** CRP ≥ 66.4 mg/L had sensitivity of 71.6%, specificity 72.5% in predicting APN | **1)** - QUADAS scale: Yes for 11/13 elements - - CRP is not reliable in diagnosing UTI in febrile infants <8 weeks of age  **2)** - QUADAS scale: Yes for 13/13 elements  - PCT is useful in predicting APN initially and in F/U of renal scars later.  -CRP is sensitive but not specific in predicting APN initially  **3)** - QUADAS scale: Yes for 11/13 elements  - Retrospective chart review; findings restricted to children < 2yrs with proven febrile UTI  **4)** - QUADAS scale: Yes for 11/13 elements  - Retrospective chart review; findings restricted to children with proven febrile UTI and fever > 2 days |
| **Gastroenteritis**  Maecus, et al.^[14]^  (2007) | **C-S** | Children with febrile GE, 4 days-17 yrs | **I:** QR-CRP  **C:** Blood & stool cultures | Differentiate bacterial vs nonbacterial GE | QR-CRP of ≥95 mg/L had sensitivity of 87%, specificity 91.7% in predicting positive stool culture in febrile GE | - QUADAS scale: Yes for 9/13 elements  - Findings are specific to the Quick Read-CRP test  - QR-CRP of ≥95 mg/L during the first 48 hours of febrile GE is suggestive of bacterial etiology |
| **Meningitis**  **1)** Sorumen, et al.^[25]^  (1999)  **2)** Sutinen, et al.^[26]^  (1999)  **3)** Dubos, et al.^[19]^  (2006) | **1) R-S**  **2) R-S**  **3) R-S** | **1)** Children with Gram stain-negative bacterial vs viral meningitis, age > 3m  **2)** Children with CNS infections, ages 0-16 yrs  **3)** Children hospitalized for bacterial or aseptic meningitis | **1) I:** Serum CRP & other laboratory parameters  **C:** CSF culture  **2) I:** Serum CRP  **C:** Final diagnosis based on cultures  **3) I:** Serum PCT, CRP and other laboratory markers  **C:** CSF & blood cultures | **1)** Differentiate Gram stain-negative bacterial from viral meningitis  **2)** Differentiate bacterial meningitis from other CNS infections  **3)** Distinguish bacterial from aseptic meningitis | **1)** Serum CRP ≥20 mg/L had sensitivity of 96%, specificity 93%  **2)** Serum CRP >50mg/L had a sensitivity of 94%, specificity 65%, NPV 96%  **3)** PCT ≥0.5ng/mL and CSF protein ≥0.5 g/L were the best predictors of bacterial meningitis | **1)** - QUADAS scale: Yes for 9/13 elements  - Retrospective chart review  **2)** - QUADAS scale: Yes for 9/13 elements  - Retrospective chart review; study done in a developing country prior to the era of HiB or PCV vaccination  **3)** - QUADAS scale: Yes for 9/13 elements  - Retrospective study; CRP had lower accuracy than PCT or CSF protein in predicting bacterial meningitis |
| **Osteomyelitis/Septic Arthritis**  **1)** Unkila-Kallio, et al.^[20]^ (1994)  **2)** Unkila-Kallio, et al.^[21]^ (1994)  **3)** Roine I, et al.^[22]^ (1995)  **4)** Kallio et al.^[23]^  (1997) | **1)** Case-series  **2)** Case-series  **3)** Case series  **4)** Case series | **1)** Children with bacteriologically-confirmed acute osteomyelitis, ages 2 weeks-14 yrs  **2)** Children with acute osteomyelitis with & without concurrent septic arthritis  **3)** Children with acute hematogenous osteomyelitis, ages 6.3±3.8 yrs  **4)** Children with bacteriologically-proven septic arthritis, ages 6 m-18 yrs | **1) I:** Serial CRP, ESR & WBC  **C:** Clinical course  **2) I:** Serial CRP, ESR & WBC  **C:** Clinical course  **3) I:** Serial CRP & ESR  **C:** Clinical course  **4) I:** Serial CRP, ESR & WBC  **C:** Clinical course | **1)** Evaluate the value of ESR, CRP and WBC as prognostic markers in acute osteomyelitis  **2)** Compare CRP, ESR & WBC values in acute osteomyelitis with & without concurrent septic arthritis  **3)** Investigate utility of CRP during recovery from acute hematogenous osteomyelitis  **4)** Compare CRP, ESR & WBC values in septic arthritis | **1)** CRP > 19mg/L present in 98% of cases on admission, peaked on day 2, and returned to normal in 1 week, much faster than ESR  **2)** CRP much higher on admission if concurrent septic arthritis present & increased dramatically on day 2  **3)** CRP was high on admission and decreased in response to therapy as of second day  **4)** CRP was high on admission in 95% of cases, peaked on day 2, and normalized on day 9 | **1)** - QUADAS scale: Yes for 5/13 elements  - Prospective data collection of case-series; CRP is a better diagnostic & prognostic marker than ESR in acute osteomyelitis  **2)** - QUADAS scale: Yes for 6/13 elements  - Prospective data collection of case-series;  - Doubling of CRP in acute osteomyelitits on day 2 is suggestive of concurrent septic arthritis  **3)** - QUADAS scale: Yes for 6/13 elements  - Prospective data collection of case-series;  - CRP helpful in follow up & prognostication of osteomyelitis  **4)** - QUADAS scale: Yes for 7/13 elements  - CRP is more useful than ESR in follow up of septic arthritis |
| **Acute Appendicitis**  **1)** Groselj-Grenc, et al.^[15]^ (2007)  **2)** Beltrán, et al.^[24]^ (2007) | **1)** C-S  **2)** C-S | **1)** Children hospitalized for suspected acute appendicitis 2.8-13.6 yrs  **2)** Children operated for appendicitis 2-14 yrs | **1)** **I:** IL-6 and U/S  **C:** Clinical exam, CRP, WBC, differential  **2) I:** CRP & WBC  **C:** Operative findings | **1)** To compare diagnostic accuracies of different tests in acute appendicitis vs. non-specific abdominal pain or mesenteric adenitis  **2)** To determine cutoff values of tests at different periods of disease evolution; to investigate diagnostic utility in discriminating simple from perforated appendicitis | **1)** U/S had highest diagnostic accuracy (92.9%) & CRP had lowest accuracy (63.7%)  **2)** CRP &/or WBC have high sensitivity in diagnosing acute appendicitis (90-100%), and high specificity in differentiating simple from perforated appendicitis (70-90%). | **1)** - QUADAS scale: Yes for 7/13 elements  - Prospective comparison of convenience sample of children with appendicitis vs. mesenteric adenitis or non-specific abdominal pain.  **2)** - QUADAS scale: Yes for 8/13 elements  – Convenience sample  - CRP & WBC may be helpful diagnostic tools in acute appendicitis |
| **Otitis Media**  **1)** Principi, et al.^[16]^  (1986)  **2)** Tejani, et al.^[17]^ (1995) | **1)** **C-S**  **2) C-S** | **1)** Children with AOM  1m-12 yrs  **2)** Children with AOM 3m-7 yrs | **1) I:** Serum CRP  **C:** Effusion culture  **2) I:** Serum CRP  **C:** Effusion cultures | **1)** Differentiate bacterial from viral AOM  **2)** Differentiate bacterial from viral AOM | **1)** CRP >15mg/L sensitivity 72%, specificity 33%  **2)** CRP >2mg/L was present in 22% of bacterial vs 6% in nonbacterial AOM. | **1)** - QUADAS scale: Yes for 11/13 elements; Valid study  - CRP is of low accuracy in differentiating bacterial from viral AOM  **2)** - QUADAS scale: Yes for 13/13 elements  - CRP is not helpful in discriminating bacterial from viral AOM |

I: Intervention; C: comparison intervention; AOM: acute otitis media; APN: acute pyelonephritis; CNS: central nervous system; CSF: cerebrospinal fluid; CXR: chest X-ray; C-S: cross-sectional; GE: gastroenteritis; IL-6: interleukin-6; LR^+^: positive likelihood ratio; LR^-^: negative likelihood ratio; NPV: negative predictive value; PCT: procalcitonin; QR: Quick-Read; R-S: retrospective study; SR: systematic review; UTI: urinary tract infection; U/S: ultrasound; HiB: Haemophilis influenza type B; PCV: pneumococcal conjugate vaccine.
