# Supplementary material for: Impact of C-reactive protein test results on evidence-based decision-making in cases of bacterial infection
Source: BMC Pediatr. 2012 Sep 3;12:140. doi: 10.1186/1471-2431-12-140 (PMC3457842; doi:10.1186/1471-2431-12-140)
Supplement: Additional file 2 — APPENDIX II.Summary of studies on the utility of CRP in neonatal sepsis. [file 1471-2431-12-140-S2.doc]

**APPENDIX II:** Summary of studies on the utility of CRP in neonatal sepsis.

| **Study author (Year)** | **Subjects** | **Interventions** | **Outcomes** | **Findings** | **Quality & Comments** |
| --- | --- | --- | --- | --- | --- |
| **Systematic Reviews**  Da Silva, et al.^[4]^ (1995)  Fowlie, et al.^[9]^  (1998) | Neonates with neonatal sepsis; NICU setting  Infants <90 days with suspected bacterial infection | **I:** Serum CRP, leukocyte indices  **C:** Reference standard of microbiologic diagnosis  **I:** Common diagnostic tests for bacterial infection, including single or serial CRP  **C:** Microbiologic or radiologic reference standard | Accuracy, sensitivity, and specificity in diagnosing neonatal sepsis  Accuracy, sensitivity, specificity, predictive values, and likelihood ratios of different tests in diagnosing bacterial infection | CRP: 15 studies were included, significant heterogeneity existed, variable range of sensitivities and specificities were found.  Leukocyte indices: 4 studies were included with significant heterogeneity and variable range of sensitivities and specificities  For single or serial CRP: Variable sensitivities, specificities, predictive values, and likelihood ratios were found. Studies were of poor methodological quality. CRP was of limited value in diagnosing infection | -Valid review; 15/16 studies had high quality score of ≥7/9.  - Because of significant heterogeneity and variable ranges of sensitivity & specificity, CRP or leukocyte indices are unreliable as single predictors of sepsis  Search was limited to Medline database only |
| **Randomized controlled trial**  Ehl, et al.^[29]^  (1997) | Neonates >1500 gms in low and intermediate care nursery and receiving antibiotics, excluding those with central catheters, mechanical ventilation, meningitis, wounds, or chromosomal anomalies | CRP measured at baseline and at 24-48 hours, and neonates divided into 3 groups according to CRP level at 24-48 hours:  **C:** infection unlikely group, CRP<10mg/l, antibiotics stopped at 24-48 hours  **I:** infection likely, CRP>10 mg/l, divided into 2 groups with stratified randomization according to CRP< or >25mg/l:  **a)** CRP-guided therapy with daily CRP measurement and antibiotics stopped when CRP<10mg/l  **b)** 5-day antibiotic therapy with CRP measured at day 5 and antibiotics stopped if <10mg/l | CRP NPV, relapse rates, treatment duration | NPV of CRP for further treatment was 99% (95%CI: 95.4%, 99.9%). CRP-guided therapy group had shorter duration of antibiotic therapy (3.7 vs 5.5 days) | -A pilot study with small sample size, and per protocol analysis.  -Reference standard not clearly described  -CRP was used as a tool to diagnose the condition  -CRP <10mg/l after at least 24 hours of antibiotics may be helpful as a guide to discontinue treatment. Findings are not generalizable to neonates with meningitis, mechanical ventilation, central catheters, wounds or chromosomal anomalies |
| **Cross-sectional studies**  Ng, et al.^[28]^  (1997)  Chan, et al.^[27]^  (1997)  Benitz, et al.^[30]^  (1998)  Couto, et al.^[31]^  (2007) | VLBW neonates with suspected LOS (>72 hours); VLBW well controls  VLBW neonates with suspected sepsis  Neonates with suspected sepsis  Neonates with blood culture-positive LOS treated with antibiotics excluding catheter-related and site-specific infections; historical controls with blood culture-proven LOS treated for 14 days with antibiotics | **I:** Serial measurements of serum IL6, TNF-alpha, IL-1, CRP, E-selectin  **C:** Microbiologic or radiologic reference standard  **I:** Serum CRP, WBC count, ANC count, platelets count  **C:** Clinical signs of sepsis and a positive culture  **I:** Serial CRP  **C:** Microbiologic or radiologic reference standard    **I:** Serial CRP every 2 days with antibiotic stopped when CRP≤12mg/l and resolution of all clinical signs  **C:** Historical controls | Optimal cutoff values, sensitivity, specificity, and predictive values for each test and for combination of tests in predicting systemic infection  ROC, Sensitivity, specificity and predictive values  ROC, Sensitivity, specificity and predictive values  Length of antibiotic therapy; mortality and relapse rates | Optimal cutoff for CRP was 12 mg/dl; IL-6 had highest sensitivity and NPV at baseline; CRP was best single marker at 24-48 hours ; Best predictor was for combination of IL-6 and CRP at baseline with either TNF-alpha on day 1 or CRP on day 2 (sensitivity 98%, specificity 91%)  Optimal CRP cutoff was 0.7 mg/dl; sensitivity 56%, specificity 72%, NPV 57%. WCC and ANC had lower predictive accuracy than CRP  Three serial CRP levels had high sensitivities for proven or probable sepsis (97.8%-98.1%) with high negative predictive values (99.7%-98.7%)  Intervention group had less days of antibiotic therapy (9 vs 16 days, p<0.001); Similar rates of relapsing sepsis and overall mortality in both groups | - QUADAS scale: Yes for 12/13 elements; Valid study  - Findings restricted to VLBW neonates with LOS  - QUADAS scale: Yes for 12/13 elements  - Single value of CRP>0.7 mg/dl is a poor predictor of sepsis  - QUADAS scale: Yes for 12/13 elements  - Serial CRP is useful in diagnostic evaluation of sepsis  - QUADAS scale: Yes for 5/13 elements  - No reference standard to compare with CRP  - CRP used as a marker to guide length of treatment  - Serial CRP may be helpful in guiding therapy; findings are applicable to culture-proven LOS (not catheter-related or site specific) |

VLBW: very low-birth weight; LOS: late-onset sepsis; WBC: white blood cells; ANC: absolute neutrophil count; gms: grams, NPV: negative predictive value.
